# Supplementary figures and images for: Mucuna pruriens Administration Minimizes Neuroinflammation and Shows Anxiolytic, Antidepressant and Slimming Effects in Obese Rats
Source: Molecules. 2020 Nov 26;25(23):5559. doi: 10.3390/molecules25235559 (PMC7730813; doi:10.3390/molecules25235559)

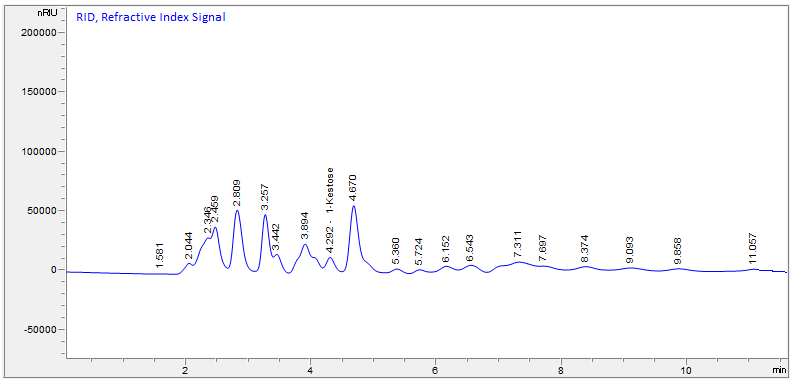

Supplement: Supplementary file 1 [file molecules-25-05559-s001.zip › Supplementary materials/Figure S1.tif]

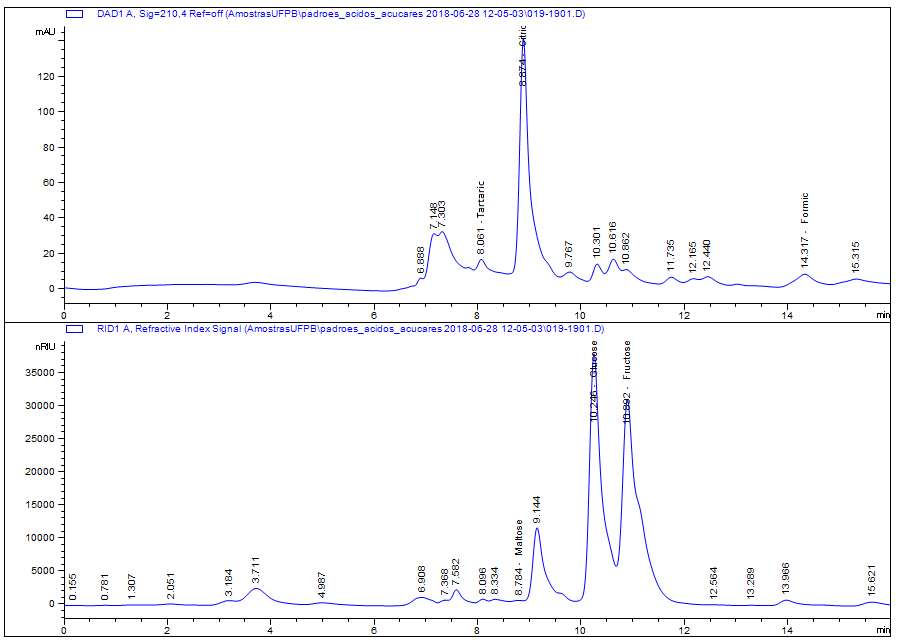

Supplement: Supplementary file 1 [file molecules-25-05559-s001.zip › Supplementary materials/Figure S2.tif]

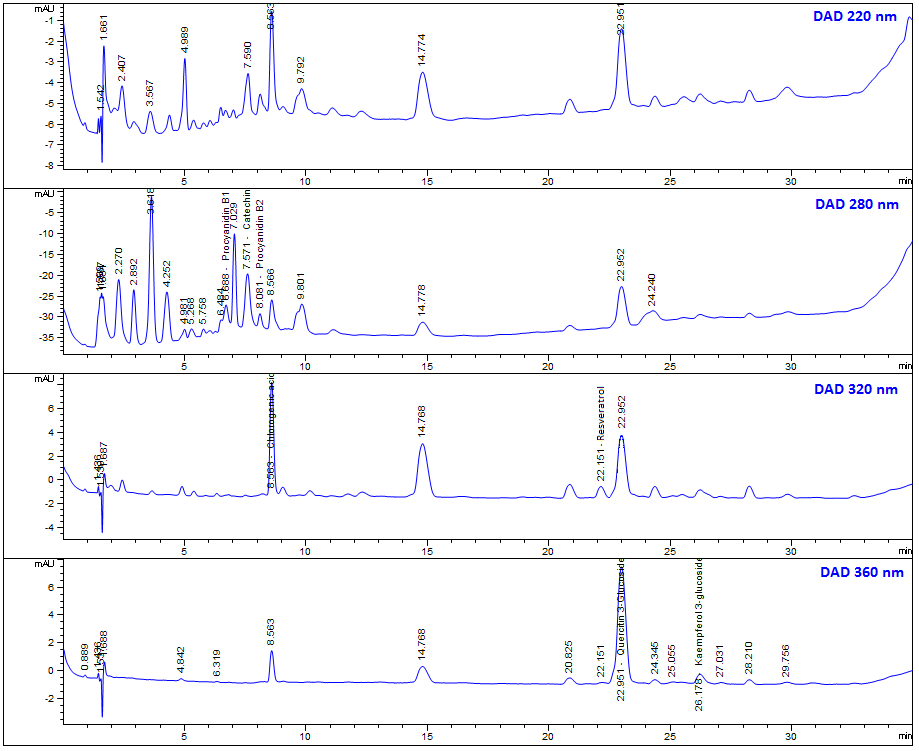

Supplement: Supplementary file 1 [file molecules-25-05559-s001.zip › Supplementary materials/Figure S3.tif]

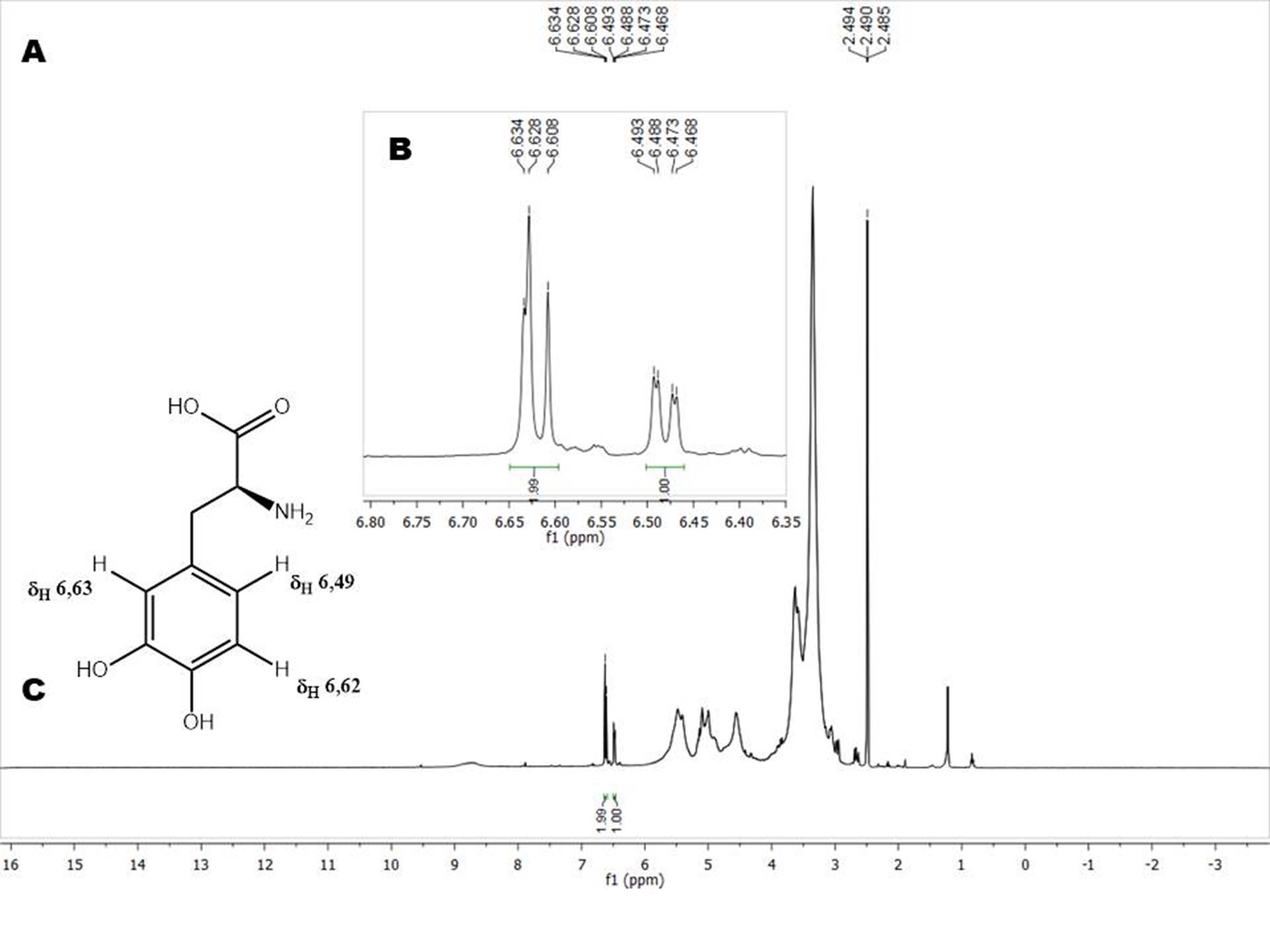

Supplement: Supplementary file 1 [file molecules-25-05559-s001.zip › Supplementary materials/Figure S4.jpg]

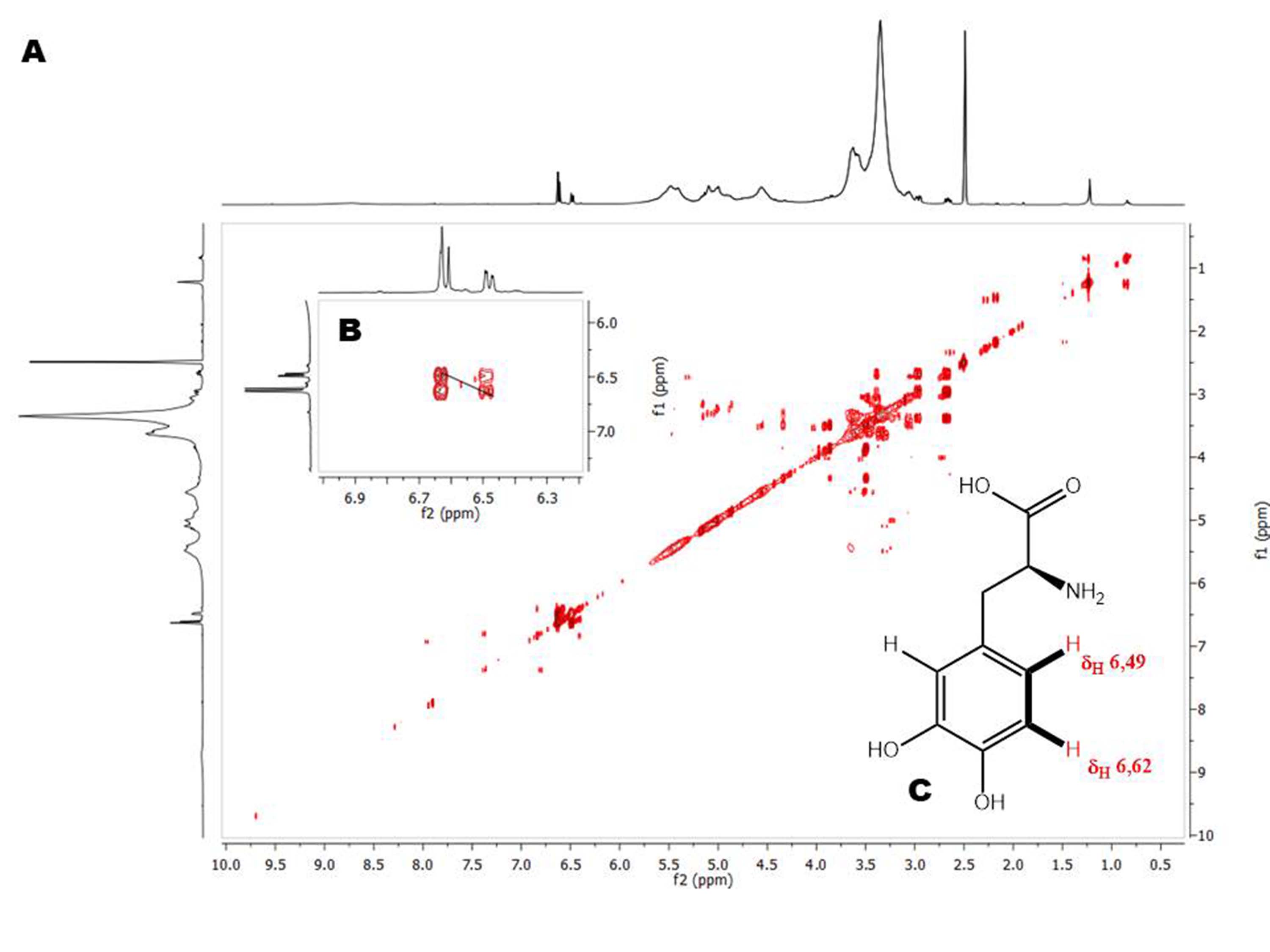

Supplement: Supplementary file 1 [file molecules-25-05559-s001.zip › Supplementary materials/Figure S5.jpg]
